# Essential components in natriuretic peptide-guided management of heart failure: an intervention synthesis

**DOI:** 10.1136/openhrt-2018-000826

**Published:** 2018-10-08

**Authors:** Jason Oke, Alison Clements, Julie McLellan, Clare Bankhead, Clare J Taylor, Graeme Spence, Amitava Banerjee, Rafael Perera

**Affiliations:** 1 Nuffield Department of Primary Care Health Sciences, University of Oxford, Oxford, UK; 2 Farr Institute of Health Informatics Research, University College London, London, UK

**Keywords:** heart failure, natriuretic peptides, monitoring, systematic review

## Abstract

**Aim:**

To identify the key components of natriuretic peptide (NP)-guided treatment interventions which reduced hospitalisation in patients with heart failure (HF).

**Methods and results:**

We extracted detailed information on the components of interventions from studies of NP-guided treatment of HF identified in a previous systematic review. We used meta-regression techniques to assess univariate associations between components and the strength of the reduction in HF hospitalisations and all-cause mortality. A Bayesian meta-analysis approach was used to re-estimate study-level effects in order to identify the study with the most effective NP-guided monitoring intervention. Finally, we examined the intervention options common to the studies in which the 95% credible interval excluded no effect. We identified eight components of NP-guided treatment from ten studies. Univariate comparisons produced mainly equivocal results, but single trial choice and common components analysis led to similar conclusions. Using a predefined treatment protocol, setting a stringent NP target (N-terminal pro-B-type natriuretic peptide of 1000 pg/mL or B-type natriuretic peptide 100 pg/mL) and including a relative targetwere potential key components to reducing HF hospitalisations using NP-guided therapy.

**Conclusion:**

This analysis provides a description of the key components of NP-guided treatment which could help policy makers develop specific recommendations for HF management. Our research suggests that NP-guided interventions could be simplified, but more research in relevant health settings, such as primary care, is required.

**Key questions**

What is already known about this subject?

- While a comprehensive systematic review and meta-analysis had shown that the use of natriuretic peptide (NP) can reduce the risk of hospitalisation, specific guidance on which form of intervention could be adopted in practice was missing.

What does this study add?

- Our analysis highlights which components of NP-guided monitoring of heart failure may be essential in reducing heart failure hospitalisation.

How might this impact on clinical practice?

- Our analyses have helped reveal which components of monitoring are perhaps unnecessary and which components may be essential in reducing admissions for patients with heart failure.

## Introduction

B-type natriuretic peptide (BNP) and the biologically inactive N-terminal fragment (NT-proBNP) are cardiac hormones collectively known as natriuretic peptides (NPs) that are synthesised and secreted in the ventricular myocardium.^1^ Serum concentrations are related to left ventricular filling pressures, and can be useful to discriminate between heart failure (HF) and other causes of breathlessness.^2^ Concentrations are higher in untreated or decompensated HF and fall after treatment.^3^ Therefore, NPs are a useful marker of cardiac function and treatment response.

Monitoring NP concentrations may guide decisions about when to alter the dose of medication,^4^ but evidence on the benefit of guiding therapy using plasma concentrations of NP has been conflicting.^3 5^ The most recent systematic review of 18 randomised controlled trials of NP-guided treatment of HF reported no evidence on reduction in all-cause mortality nor HF mortality,^6^ but did find evidence that NP-guided treatment reduced the number of hospital admissions. As with many systematic reviews, these studies implemented closely related but not identical versions of NP-guided treatment. Therefore, it is not clear which version should be implemented in clinical practice, where in the patient pathway the test should be conducted or which features of the intervention are critical for effectiveness. Current methods to guide the transition from research evidence to clinical practice are poorly developed, and clinical guidance is often not covered in the systematic review literature.^7^ One methodological approach to overcome the challenges of moving from evidence to practice is to use an intervention synthesis as described by Glasziou *et al*.^8^ This consists of three broad approaches: (1) choosing a single trial based on criteria such as feasibility, cost and effectiveness; (2) common components hybrid which extracts and recombines components based on frequency and importance; and (3) model-guided synthesis, an approach which models the mechanisms of action to assess the importance of the intervention components. The single trial-based choice and common components hybrid method is viable for a wide range of systematic reviews, whereas the model-guided synthesis is probably only possible for large systematic reviews.^8^

The aim of this study was to determine the effective intervention components which were associated with a reduction in hospitalisations for patients with HF. This could enable policy makers and practitioners to determine specific recommendations for monitoring HF.

## Methods

Our intervention synthesis builds on a systematic review of the effectiveness of NP-guided monitoring for HF.^6^ In brief, the systematic review included trials comparing management guided by serial BNP or NT-proBNP with usual care. The primary outcome in the original review was all-cause mortality with secondary outcomes of HF mortality, HF admissions, adverse events, costs and quality of life. For our intervention synthesis, we used HF hospitalisations as the primary outcome and all-cause mortality as a secondary outcome. All-cause mortality was considered as a secondary outcome because in the original systematic review NP-guided therapy was not associated with a significant reduction in all-cause mortality, whereas it was associated with a significant reduction in HF hospitalisations.

We contacted authors to obtain information regarding the interventions evaluated as part of the systematic review.^6^ For the purpose of the intervention synthesis, we went back to the original trial publications and attempted to gather as much detail on the components of intervention as possible. Two members of the team (AC and CB) extracted and coded the component information.

First, we examined univariate associations between each component and size of the relative risk (RR) reduction by comparing effect estimates in subcategories of each component. We compared the RRs of hospitalisation and all-cause mortality across component subgroups, using meta-regression to test for equality.^9^ We presented the estimated ratio of RRs with 95% CIs for each component in a figure. For defining stringency of the target, we used the lowest threshold reported in the included studies and defined stringency as 100 pg/mL (or equivalent units) for BNP and 1000 pg/mL for NT-proBNP. We converted targets in units of pmol/L for NT-proBNP using an online calculator^10^ and assumed that ng/L and pg/mL were equivalent (ie, 100 ng/L is the same as 100 pg/mL). We report thresholds in units of pg/mL because the majority of the studies in the review reported NP in this unit. Studies with higher targets were classed as not stringent. We looked at the frequency of monitoring across the whole length of the study (ie, titration and control phase). Studies were deemed to be ‘more frequent’ monitoring if at any time scheduled visits were at intervals less than every 3 months.

We then re-estimated the study-level RRs with 95% credible intervals using a Bayesian meta-analysis approach (see online supplementary file 1). This was to account for biases in which smaller studies report larger effect sizes.^11^

10.1136/openhrt-2018-000826.supp1 Supplementary data

We first identified the trial with the largest RR reduction (single-trial choice) following re-estimation and assessed this version of the intervention. We then separated the trials into studies that appeared to have the ‘more effective’ versions of the intervention using the criteria of the 95% credible interval. The ‘more effective’ studies had a 95% credible interval which excluded ‘no difference’ (RR=1), whereas in the ‘less effective’ studies the 95% credible interval crossed the line of ‘no difference’. Component options were selected for the new composite intervention based on which of the component choices were more common to the ‘more effective’ studies with any ties considered equivocal.

## Results

The review found 3394 references and identified 18 studies suitable for consideration. One further study has been published since the review was finished.^12^ HF hospitalisation rates were reported in 11 studies: Troughton *et al*,^3^ Jourdain *e* *t al*,^13^ Anguita *et al*,^14^ Berger *et al*,^15^ Krupicka *﻿et al*,^16^ Lainchbury *et al*,^17^ Karlström *et al*,^18^ Januzzi *et al*,^19^ Schou *et al*,^20^ Skvortsov 2015^21^ and Felker *et al*.^12^ Mortality outcomes were reported in six additional studies: Pfisterer *et al*,^22^ Beck-da-Silva *et al*,^23^ Persson *et al*,^24^ Eurlings *et al*,^25^ Shah *et al* 26 and Shochat *et al*.^27^ Hospitalisation outcomes but not mortality were reported in Januzzi *et al*,^19^ and hence 16 studies were included in the secondary analysis. A flow chart showing the selection process can be found in online supplementary file 1.

We identified eight components of NP-guided treatment from the original ten studies (table 1).

**Table 1:** List of components extracted

Component	Description	Categorisation (where applicable)
1. Setting	Where the majority of the patients’ management took place, that is, specialist outpatient or at ambulatory clinics.	Specialist heart failure or cardiology clinic. Non-specified outpatient or other.
2. Telephone contact	Was telephone contact with a healthcare professional available to patients?	Yes. None specified.
3. Education	Did the studies incorporate education for self-management?	Yes. None specified.
4. Treatment protocols	Did the studies use predefined rules/algorithms for the uptitration of medication?	Predefined. Investigator judgement.
5. Targets	Was the target NP level based on an absolute level or were relative changes to baseline considered?	Absolute. Incorporated relative changes.
6. Stringency	How aggressive was the NP target?	Less stringent.* Stringent.
7. Trigger	Was a relative value set for treatment changes (eg, reduction by 30%)?	Yes. No.
8. Monitoring frequency	What was the most frequent rate of monitoring (either in titration or control phase)?	More frequent than every 3 months. Every 3 months or longer

*Threshold for less/more stringent was 100 pg/ml (or equivalent units) for BNP and 1000 pg/ml for NT-proBNP.

NP, natriuretic peptide.

An evidence summary table that shows detailed descriptions of the specific components used for each study can be found in online supplementary table S1.

### Components and univariate associations with hospitalisation and all-cause mortality outcomes

#### Component 1: setting

Twelve of the studies were based in specialist HF or cardiology clinics. In four studies, the descriptions were ambiguous or unclear, and so were classed as non-specified or other.

#### Component 2: telephone contact

Only three studies in total included telephone contact as part of the intervention. In the Berger *et al* 15 study, both the multidisciplinary care and NT-proBNP-guided intensive management arms included telephone contact with an HF nurse. Similarly, the patients enrolled in the Schou *et al* 20 study had free daily access to telephone consultations with an HF nurse who was supervised by cardiologists.

#### Component 3: education

Six studies included some form of an educational component to their intervention. In some studies, education was aimed at promoting adherence to medication, whereas in others education was broader and promoted enhancement of self-management and lifestyle.

#### Component 4: treatment protocols

Eight studies used predefined (but different) treatment protocols which laid out specific instructions for uptitration of therapy. Six studies used investigator judgement to modify therapy in response to NP monitoring. The Januzzi *et al* 19 study justified this choice by stating that ‘it was believed that such an approach would confound the concept of standard HF, which does not typically rely on such care’. In two of the studies, it was not possible to ascertain if a predefined or investigator judgement approach was used.

#### Component 5: NP targets

Nine studies set goals based on absolute target NP levels, although these varied in stringency. Six studies included a relative NP target based on baseline NP level. For example, Skvortsov *et al* 21 set a goal of getting NT-proBNP below 1000 pg/mL or 50% lower than the baseline NT-proBNP level. Schou *et al* 20 did not set a target.

#### Component 6: target stringency

In all but one study, a single target was set. Karlström *et al* 18 defined BNP targets of 150 ng/L in patients <75 years and 300 ng/L (BNP) target for patients over 75. Five studies set ‘stringent’ targets and ten studies set ‘less stringent’ targets. As Schou *et al* 20 did not set a target, we did not include this study in this comparison.

#### Component 7: NP trigger

None of the studies made no mention of using a specific trigger value of NP and set only an overall target level NP. Seven studies were classed as having set ‘Relative’ triggers to change therapy. For example, the Berger *et al* 15 study used an increase or decrease of 30% to optimise therapy. Similarly the Schou *et al* 20 study set a reference change value of 30% increase from baseline to take action and mandated completion of a clinical checklist in the absence of symptoms or signs of congestion.

#### Component 8: monitoring frequency

**Figure 1:**
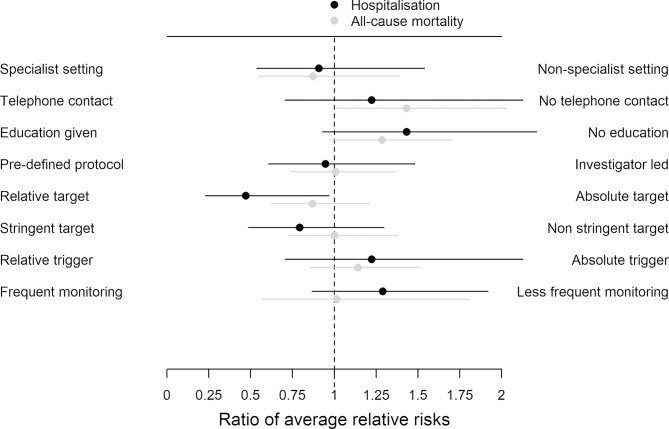
Results of the meta-regression of individual components.

Twelve studies monitored NP more frequently than every 3 months and four studies (Anguita *et al*,^14^ Jourdain *et al*,^13^ Krupicka *et al,* 16 and Lainchbury *et al* 17) set monitoring intervals at 3 months or longer (figure 1).

### Single-trial-based choice

**Figure 2:**
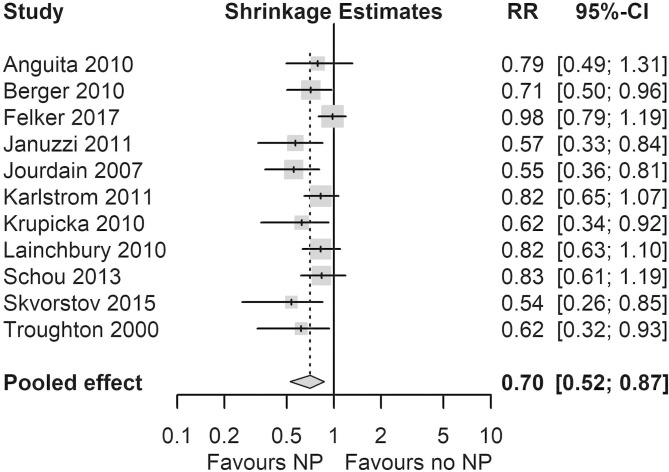
Forest plot showing ‘shrinkage estimates’ of the relative risk (RR) of hospitalisation. NP, natriuretic peptide.

The Skvortsov *et al* 21 study had the largest RR reduction in hospital admissions and all-cause mortality using standard meta-analysis methods (see online supplementary file 1), but this was a fairly small study (N=76) and the effect may be optimistic (a small study effect). The Bayesian ‘shrinkage’ analysis, which re-estimates study-level effects, pulled back the Skvortsov *et al* 21 study from an observed RR (95% CI) of 0.25 (0.09 to 0.67) to 0.54 (0.26 to 0.85) for hospitalisation and from 0.35 (0.12 to 0.98) to 0.78 (0.58 to 0.96) for all-cause mortality. Following re-estimation, the Skvortsov *et al* 21 study remained the study with the largest reduction in both hospital admissions and all-cause mortality, but shrinkage analysis had the effect of making it comparable with Januzzi *et al*,^19^ Jourdain *et al* 13 and Berger *et al* 15 (figure 2).

Patients in the Skvortsov *et al* 21 study were managed in a specialist HF clinic. The intervention did not include telephone contact or any specialist education component. At each visit, NP measurement was considered in conjunction with clinical assessment, and specific instructions were given on the basis of clinical symptoms and NP trends. Target NP levels were stringent (NT-proBNP 1000 pg/mL), but relative changes from baseline (>50% reduction) were also included. Outpatient monitoring was monthly in controlled patients and every 2 weeks in those whose clinical condition appeared to be deteriorating. A notable feature of the Skvortsov *et al* 21 study is that, unlike the other studies, the majority of the patients recruited into the study were New York Heart Association (NYHA) stage 4 (see online supplementary file 1).

### Common components approach

After re-estimation using the Bayesian approach, in the hospitalisation outcome, six studies had credible intervals excluding RR of 1 (the ‘more effective’ interventions) and five studies had credible intervals that crossed RR of 1 (considered ‘less-effective’ versions of the interventions). For all-cause mortality, seven studies had credible intervals that excluded 1 and the remaining nine had credible intervals that crossed 1. No component options were exclusive to the more effective studies (ie, occurred in all of the more effective studies and in none of the less effective studies). Applying the common components approach to the hospitalisation outcome (table 2), our proposed new composite intervention would be based in a specialist clinic, would not include an education component or provide telephone contact but would use a predefined treatment protocol, aim to reduce NP to a stringent target (<1000 pg/mL), and also consider relative targets (eg, 50% reduction from baseline) but not include a relative trigger (ie, increasing doses based on short-term changes in NP). Applying the same method to the all-cause mortality outcomes (table 3), our composite intervention would be comprised of a pre-defined treatment protocol, a relative and stringent target with more frequent monitoring. Monitoring frequency was considered equivocal for the hospitalisation outcome.

**Table 2:** HF hospitalisation: Presence or absence of component options split by studies with study-specific credible intervals excluding the null effect (RR=1) (lower part of the table) and studies with credible intervals not excluding the null effect (upper part of the table)

	Set in heart failure clinic	Telephone contact	Educational component	Predefined treatment protocol	Incorporated relative NP target	Stringent target	Relative NP trigger value	Monitoring more frequently (<every 3 months)
Studies not excluding, RR=1								
Anguita *et al* 14	✔	✗	✗	✗	✗	✔	✗	✔
Felker *et al* 12	✔	✗	✗	✗	✗	✔	✗	✔
Karlström *et al* 18	✔	✗	✔	✔	✗	✗	✗	✔
Lainchbury *et al* 17	✗	✗	✔	✗	✗	✗	✗	✗
Schou *et al* 20	✔	✔	✔	✔	NA	NA	✔	✔
✔ Proportion	0.80	0.20	0.6	0.40	0	0.5	0.20	0.80
Studies excluding, RR=1								
Berger *et al* 15	✔	✔	✔	✔	✗	✗	✔	✔
Januzzi *et al* 19	✔	✗	✗	✗	✗	✔	✗	✗
Jourdain *et al* 13	✗	✗	✗	✗	✗	✔	✗	✗
Krupicka *et al* 16	✔	✗	✗	?	✔	✔	✗	✗
Skvortsov *et al* 21 2015	✔	✗	✗	✔	✔	✔	✗	✔
Troughton *et al* 3	✔	✗	✗	✔	✗	✗	✗	✔
✔ Proportion	0.833	0.167	0.167	0.6	0.33	0.667	0.167	0.5
Proposed new composite intervention	**✔**	**✗**	**✗**	**✔**	**✔**	**✔**	**✗**	–

Component options for new composite intervention are selected based on the most common option in the ‘more effective’ studies.

NA, not available;NP, natriuretic peptide;RR, relative risk.

Component options based on all-cause mortality were the same as for the hospitalisation outcome, with the exception of monitoring frequency. For all-cause mortality, the studies that monitored more frequently than every 3 months outnumbered the studies monitoring less frequently. Only the Skvortsov *et al* 21 study included all of these specific component options.

## Discussion

In our study we have attempted to identify the key components of NP-guided monitoring for patients with chronic HF using an intervention synthesis approach. While a comprehensive systematic review and meta-analysis had shown that the use of NP can reduce the risk of hospitalisation, specific guidance on which form of the intervention could be adopted in practice was missing. Our analyses have helped reveal which components are perhaps unnecessary and which components may be essential in reducing admissions for patients with HF.

The Bayesian meta-analytic approach had the effect of reducing heterogeneity in the all-cause mortality outcome, suggesting that most of the observed variation is attributable to sampling variability as opposed to ‘real’ treatment effect modification. For HF hospitalisation, the heterogeneity was preserved after the Bayes analysis, suggesting the observed differences in intervention efficacy may be real. While univariate analyses of individual components produced mainly equivocal results, the single trial-based choice and common components analysis led to a similar conclusions; using a predefined treatment protocol and setting a stringent target alongside a relative target are potentially key components in NP-guided therapy reducing HF hospitalisations.

Current guidelines from the European Society of Cardiology (ESC)^28^ highlight the residual uncertainty about the value of NP-guided therapy and make no specific recommendations for monitoring. The National Institute for Health and Care Excellence suggests considering specialist monitoring of patients in whom uptitration is problematic or who have been admitted to the hospital.^29^ A recent individual patient data meta-analysis reported that the benefit of NP-guided therapy is confined to patients with heart failure with reduced ejection fraction (HFrEF), and although comorbidities were associated with mortality outcomes this was not the case for HF-related hospitalisations.^30^ Similarly, a meta-regression found limited evidence of effect modification around therapy types, NYHA class, and having hypertension or diabetes, but length of follow-up and age were associated with HF-related hospitalisations.^31^

Cocco *et al* 32 also did examine why only some of the many NP-guided trials demonstrated encouraging trends. They concluded that the different approaches used in the studies with particular respect to patient selection (acute vs chronic forms of HF) and the choice of NP target were important. Some of the studies examined, set targets that were too high to reduce cardiac events, which is consistent with our finding that stringency of target may be associated with reducing hospitalisation. Finally, they observed that target NP values were chosen empirically, as opposed to being evidence-based, and these levels varied significantly. As per the ESC position they also concluded that NP-guided therapy usually led to intensification of therapy,^32^ and this could potentially explain reductions in HF-related hospitalisations. We could not rule this out with the data available to us.

The results of this analysis can be generalised to a diverse patient group; all of the studies included people of older age (up to 85 years of age), while some but not all of the studies recruited patients with heart failure with preserved ejection fraction (HFpEF) as well as HFrEF. There were also patients represented from a wide spectrum of chronic HF, including asymptomatic and symptomatic forms of the disease, but not those with significant comorbidities, awaiting heart surgery or low life expectancy, who were excluded. Finally, the interventions were for the most part well described, and as part of the initial systematic review detailed information on the interventions used in the trials was obtained.

There are a number of limitations to our analysis. The initial systematic review only identified 10 studies that reported HF hospitalisation rates and 15 with all-cause mortality outcomes. Even with the addition of the most recent trial,^12^ this may be too few to be able to identify all of the ‘active’ components of most interventions. The small sample size is likely to have limited our ‘statistical power’ to detect all but the largest treatment covariate interactions. The studies that have evaluated the use of NP-guided monitoring in patients with HF are heterogeneous for factors other than the intervention components examined in this study. Therefore, a more nuanced analysis that is able to adjust for the varying demographic and clinical characteristic of the participants, outcome evaluation and assay techniques may be required to determine the optimal approach to NP-guided monitoring. This is probably only possible with a comprehensive individual patient data meta-analysis including all of the relevant studies.

By doing multiple comparisons in the univariate analyses, we run the risk that a statistical difference could occur by chance. In fact, only 1 of the 16 comparisons we made was statistically significant at the 5% level, and it therefore seems more likely that with this number of studies we are more likely to be at risk of making a type 2 error and missing real differences. In addition, the outcome does not take account of how long these hospital stays were and this could also have been important. In all of the studies contributing hospitalisation data, NP was managed in ambulatory clinics by HF specialists, and therefore does not provide any evidence that NP-guided monitoring if implemented in a primary care setting could reduce hospitalisation to the same degree. Berger *et al* 15 warned that ‘deployment of their intervention using ambulatory HF specialists and home nurses may not be feasible in all health care systems, and further adaptation of this approach will be necessary in different settings’. Our analysis did not extend to considering monitoring with BNP versus NT-proBNP because in the initial review a stratified analysis comparing NT-proBNP with BNP with respect to all-cause and HF mortality, all-cause and HF admissions and quality of life showed no differences.^6^ However, there is evidence to suggest that BNP immunoassays exhibit greater between-method variability than NT-proBNP assays (coefficient of variation (CV) for BNP=43.0%, CV for NT-proBNP=8.7%), suggesting that BNP immunoassays are affected by large systematic differences in analytical performance.^33^ On this evidence alone, one would favour NT-proBNP if NP-guided monitoring included setting a stringent target.

Lastly, adverse events were reported in only a few studies, and we have not been able to assess whether any specific version of the intervention is associated with more or fewer problems.

In this analysis we have attempted to provide information about the critical features of NP-guided monitoring of chronic HF to reduce hospitalisation. While there are tools to aid the evaluation of complex interventions in trials,^34^ when it comes to meta-analysis we are reductionist and tend to focus on simple questions which may not translate to clinical practice. The approach used in this paper takes account of the complexity of NP-guided management (or therapy) as an intervention and attempts to provide practical recommendations for monitoring. While the findings of this review were unable to unequivocally determine an optimal intervention, a future intervention synthesis incorporating more data may provide clear procedural details of the essential elements of NP-guided monitoring. Further research to evaluate the clinical and cost-effectiveness of any implemented NP-guided HF monitoring within different healthcare systems is also needed.

**Table 3:** All-cause mortality: presence or absence of component options split by studies with study-specific credible intervals excluding the null effect (RR=1) (lower part of the table) and studies with credible intervals not excluding the null effect (upper part of the table)

	Set in heart failure clinic	Telephone contact	Educational component	Predefined treatment protocol	Incorporated relative NP target	Stringent target	Relative NP trigger value	Monitoring more frequently (<every 3 months)
Studies not excluding, RR=1								
Anguita *et al* 14	✔	✗	✗	✗	✗	✔	✗	✔
Beck-da-Silva *et al* 23	✔	✗	✗	✔	✔	✗	✔	✔
Berger *et al* 15	✔	✔	✔	✔	✗	✗	✔	✔
Karlström *et al* 18	✔	✗	✔	✔	✗	✗	✗	✔
Krupicka *﻿et al* 16	✔	✗	✗	?	✔	✔	✗	✗
Lainchbury *et al* 17	✗	✗	✔	✗	✗	✗	✗	✗
Persson *et al* 24	✗	✗	✗	✗	✔	✗	✔	✔
Schou *et al* 20	✔	✔	✔	✔	NA	NA	✔	✔
Shah *et al* 26	✔	✔	**﻿✔**	✗	✗	✗	✔	✔
✔ Proportion	0.778	0.333	0.555	0.5	0.375	0.25	0.555	0.778
Studies excluding, RR=1								
Eurlings *et al* 25	✔	✗	✔	✔	✔	✗	✔	✔
Felker *et al* 12	✔	✗	✗	✗	✗	✔	✗	✔
Jourdain *et al* 13	✗	✗	✗	✗	✗	✔	✗	✗
Pfisterer *et al* 22	✔	✗	✗	✔	✗	✗	✗	✔
Shochat *et al* 27	✗	✗	✗	?	✔	✗	✔	✔
Skvortsov *et al* 21	✔	✗	✗	✔	✔	✔	✗	✔
Troughton *et al* 3	✔	✗	✗	✔	✗	✗	✗	✔
✔ Proportion	0.714	0	0.142	0.667	0.429	0.429	0.286	0.857
Proposed new composite intervention	✗	**✗**	**✗**	**✔**	**✔**	**✔**	**✗**	**✔**

Component options for the new composite intervention are selected based on the most common option in the ‘more effective’ studies.

NA, not available;NP, natriuretic peptide;RR, relative risk.
