# Supplementary material for: Essential components in natriuretic peptide-guided management of heart failure: an intervention synthesis
Source: Open Heart. 2018 Oct 8;5(2):e000826. doi: 10.1136/openhrt-2018-000826 (PMC6196949; doi:10.1136/openhrt-2018-000826)
Supplement: Supplementary data [file openhrt-2018-000826supp001.docx]

## Supplementary files

## Table S1: Evidence table summarising component options used in NP-guided treatment interventions

|  | **Setting** | **Telephone contact** | **Education** | **Treatment protocols** | **Target (Absolute vs relative)** | **NP Target stringency** | **Relative NP Trigger?** | **Monitoring frequency** |
| --- | --- | --- | --- | --- | --- | --- | --- | --- |
| **Anguita 2010** | **HF or cardiology clinic**: A single cardiology department of a Spanish hospital | **None specified** | **None specified** | **Investigator led:** “The adjustment of treatment could include several changes in each visit according to the judgment of the responsible physician, but always following the pattern deviating only if adverse effects are encountered“ | **Absolute:** Therapy was adjusted to obtain a clinical score < 2 in the symptom-guided group and BNP levels < 100 pg/ml in the BNP-guided group. | **Stringent:**  “If BNP levels were higher than 100 pg/ml the pharmacological treatment was increased“ | **No.** if BNP levels were higher than 100 pg/ml the pharmacological treatment was increased | **More frequent:** All patients were seen at 1, 2, 3, 6, 12 and 18 months. |
| **Beck-da-Silva 2010** | **HF or cardiology clinic**: Eligible patients were selected from the Heart Function Clinic from July 2002 to February 2003 | **None specified** | **None specified** | **Study-specific:** “On each visit, patients fell into one of four scenarios: 1. Clinically better, BNP decreasing: β blocker increased one step;  2. Clinically same or mildly worse, BNP decreasing: β blocker increased one step;  3. Clinically same or better, BNP increasing: β blocker unchanged;  4. Clinically worse, BNP increasing: β blocker decreased one step or discontinued. | **Relative:** If the patient presented with clinical deterioration as defined above and the serum BNP measurement increased from previous measurement, then the dose of β blocker was not increased. However, if the patient presented mild signs of congestion, such as peripheral edema, and the BNP level was more than 10% lower than the previous value, then the dose of β blocker was increased. If BNP was within 10% above or below the previous level, it was considered | **Less stringent:** No end-point target set see left panel | **Yes:**  if the patient presented mild signs of congestion, such as peripheral edema, and the BNP level was more than 10% lower than the previous value, then the dose of β blocker was increased. If BNP was within 10% above or below the previous level, it was considered unchanged and clinical signs were primarily considered as a guide to β-blocker up-titration. | **More frequent:** Patients were followed every month for 3 months by clinical assessment and measurement of BNP concentration |
| **Berger 2010** | **HF or cardiology clinic**: Mix of ambulatory visits at a CHF specialist and home visits by specialised CHF nurse. | **Yes**: Telephone contact with CHF nurse, averaged 3 contacts per person | **Yes:** Individualised patient and caregiver education and enhancement of self- management given by CHF nurse | **Pre-defined:**  “medical therapy was intensified according to a stepwise protocol. “The HF specialist used NT-proBNP in addition to other clinical and laboratory parameters for integrated clinical management (e.g., adaptation of diuretic regimen, rate of dose increase for neurohormonal antagonists, schedule of visits) according to the recommendations given in Figure 1”. | **Absolute** | **Less stringent**. A single NT-proBNP target was used. (2200 pg/ml)  Justification: The lower of two thresholds used in previous study to stratify risk of predicted HF hospitalisation The target of 2200 was derived from multiplying the 350 pg/ml(BNP) threshold by 9 and subtracting 30% | **Yes:** Trigger based on known intra-individual variability of 30%. In patients with an ongoing elevated NT-proBNP > 2,200 pg/ml, the every-2-weeks visits were continued until maximal recommended or tolerated dosages of CHF therapy were established, | **More frequent:** Every two weeks in uncontrolled patients.  Every three months if below target |
| **Eurlings 2010** | **HF or cardiology clinic**: Follow-up visits were performed by dedicated HF cardiologists and nurses. | **None specified** | Yes: A therapy advisor, incorporated in the electronic case record form, was designed to give individual treatment advice, depending on several individual variables including the cause of HF (ischemic vs. nonischemic), left ventricular ejection fraction (LVEF), clinical signs of HF, and creatinine clearance. | **Pre-defined:**  Treatment in the NT-proBNP–guided group was guided  by the combination of clinical assessment and NT-proBNP levels. The individual NT-proBNP target value was set at the lowest level at discharge or at 2 weeks follow-up. If at subsequent outpatient visits, NT-proBNP levels were more than 10% with a minimum of 850 pg/ml above this individual target level, NT-proBNP level was considered “off-target,” and therapy was intensified according to the ESC HF treatment guidelines | **Relative:** The individual NT-proBNP target value was set at the lowest level at discharge or at 2 weeks follow-up. | **Less Stringent**. Unclear (but suggest coding as less stringent as individual targets were set) | **Yes:** NT-proBNP levels were more than 10% with a minimum of 850 pg/ml above this individual target level, NT-proBNP level was considered “off-target,” and | **More frequent:** Regular follow-up visits were scheduled at 2 weeks and 1 month, and then every 3 months until the follow-up period of 2 years was completed. |
| **Felker 2017** | **HF or cardiology clinic:** 45 academic and community clinical sites. Study’s sites had substantial focus and expertise in HF care, | **None specified** | **None specified** | **Treating physician led: “**Specific adjustments  of therapy for individual patients were at the discretion  of the treating physician, but sites were encouraged to prioritize titration of neurohormonal antagonists over diuretics unless  there was clinical evidence of congestion or volume overload.” | **Absolute:** “titrate HF therapy to target an NT-proBNP level of less than 1000 pg/mL” | **Stringent**: “titrate HF therapy to target an  NT-proBNP level of less than 1000 pg/mL” | **No** | **More frequent:** All patients seen at 2 and 6 weeks and then every 3 months. Plus additional visit after 2 weeks if therapy adjustment for HF, these 2 weeks visits continue until no further titration is possible. |
| **Januzzi 2011** | **HF or cardiology clinic**: Clinic visits at the Massachusetts General Hospital Heart Center | **None specified** | None specified | **Investigator led:**  No algorithm for drug therapy introduction or intensification was used, as it was believed that such an approach would confound the concept of standard HF management, which does not typically rely on such algorithmic care. | **Absolute** | **Stringent:**  Sustained NT-proBNP concentrations <1,000 pg/ml | **No.** Patients with NT-proBNP concentrations > 1,000 pg/ml were considered for drug therapy intensification and/or careful reassessment of their medical programs irrespective of clinical status or perception of the presence of an optimal medical program | **Less frequent** Clinic visits every 3 months (±2 weeks) for a minimum of 6 months follow up |
| **Jourdain 2007** | **Unspecified** outpatient clinic | **None specified** | None specified | **Investigator led:** Each class of therapy could be modified according to the judgment of the investigator. | **Absolute** | **Stringent**  Target set at < 100 pg/ml | **No.** Medical therapy was increased with the aim of lowering plasma BNP levels (target <100 pg/ml) | **Less Frequent:**  Outpatient visits were scheduled every month for 3 months, then every 3 months. |
| **Karlstrom 2011** | **HF or cardiology** The study was conducted in 19 hospitals in Sweden (n ¼ 15) and Norway (n ¼ 4) by physicians experienced in managing HF | **None specified** | **Yes:** Patients were made aware of their BNP value in order to increase motivation to adhere to treatment | **Pre-defined**:  “Treatment recommendations in order to reduce elevated BNP  levels or signs/symptoms of worsening HF were suggested according to a pre-defined schedule”…. Only the “adjustment of loop diuretic dose was left to the discretion of the investigator”. | **Absolute** | **Less stringent.**  In the BNP-guided group, medical treatment was guided by BNP. The goal was to reduce BNP levels to <150 ng/L in patients aged 75 years and <300 ng/L in patients  aged ≥75 years | **No:** Therapy was increased if BNP elevated or signs or symptoms of worsening HF. | **More Frequent:** Outpatient visits were scheduled at weeks 2, 6, 10, 16, 24, 36, 48, and then every 6 months until study end |
| **Krupicka 2010** | **HF or cardiology clinic**: HF specialist outpatient clinic | **None specified** | None specified | **Not clear: “**Treatment guided also by effort to normalize plasma BNP levels” | **Relative**: “achieve the lowest values” | **Stringent**: < 100 pg/ml BNP | No | **Less frequent**: Not clear but seven visits in 2 years |
| **Lainchbury 2010** | **Other**: Qualifying patients attended a research outpatient clinic….. Patients randomly allocated to the hormone- guided (NT-proBNP) and CG groups were seen at 3-monthly intervals in a dedicated research clinic. | **None specified** | **Yes:** Both groups received instructions on monitoring weight, dietary sodium restriction, rest after diuretic administration, exercise, avoidance of licorice, nonsteroidal anti-inflammatory drugs, and alcohol, and the need for influenza vaccination. | **Investigator led:**  “For the NT-proBNP group, adjustments in medications  and additional follow-up visits were triggered by an NT-proBNP levels > 150 pmol/l and/or a heart failure score> 2.0 according to instructions by 1 investigator (J.G.L.) who did not undertake the clinical assessments” | **Absolute** | **Less stringent**  “Titration of medication aimed at reducing plasma levels of NT-proBNP to <150 pmol/l (~1,300 pg/ml)“ | **No:** Follow-up visits were triggered by a proBNP level>150 pmol/l and/or a heart failure score>2.0 according to instructions by 1 investigator | **Less Frequent:** Patients on hormone- guided (NT-proBNP) were seen at 3-monthly intervals |
| **Persson 2010** | **Other**: SIGNAL-HF was a 9 month, randomized, parallel group, single-blind, multi-centre study conducted in 45 primary care centres in Sweden  (Figure | **None specified** | **None specified for patients:**  Before the study, all investigators and their teams underwent 2–3 h of educational training about current CHF guidelines with a local cardiologist. | **Investigator led:**  Investigator prescribed CHF treatment was used in accordance with the national guidelines | **Relative:**  The treatments were to be intensified, if tolerated by the patient, until at least a 50% reduction from baseline NT-proBNP was achieved, even if not indicated by the patient’s clinical symptoms and signs | **Less stringent:**  Relative targets set | **Yes.** The treatments were to be intensified, if tolerated by the patient, until at least a 50% reduction from baseline NT-proBNP was achieved, even if not indicated by the patient’s clinical symptoms and signs | **More frequent,** Visits were at 10 days, 1 month, 3 months, 6 and 9 (from figure 1) |
| **Pfisterer 2009** | **HF or cardiology ward:** The study  was conducted at 15 centers in Switzerland and Germany by physicians experienced in managing HF | **None specified** | None specified | **Pre-defined:** predefined escalation rules simulating clinical practice to reduce either symptoms to dyspnea NYHA class of II or less (in the symptom-guided group) or N-terminal BNP levels to less than 2 times the upper limit of normal—less than 400 pg/mL in patients younger than 75 years and less than 800 pg/mL in patients aged 75 years or older—and NYHA class of II or less (in the N-terminal BNP–guided group9  ). | **Absolute** | **Less stringent**  —less than 400 pg/mL in patients younger than 75 years and less than 800 pg/mL in patients aged 75 years or older | **No.** | **More Frequent:**  Patients were fol- lowed up in the outpatient clinics of each center with prespecified visits after 1, 3, 6, 12, and 18 months. |
| **Schou 2013** | **HF or cardiology clinic**: Patients were recruited from 18 public heart failure clinics (HFCs) – assumed that care took place there | **Yes:** Patients had access to free daily telephone consultations with the HF nurse who was supervised by cardiologists | **Yes**: Education was enforced at each visit to ensure adherence. | **Pre-defined:**  We therefore designed an  NT-proBNP monitoring concept where increases of ≥30% in NT-proBNP compared with an individual NT-proBNP set-point mandated completion of a clinical checklist even if the patients did not become more symptomatic or signs of congestion were present” See appendix S3 for details of checklist | **No target NP set**  “NT-proBNP > 1000 pg/mL was used as an inclusion criterion, so high-risk patients were included, **but not as target** since the patients should receive guideline treatment based on LVEF, functional class, and QRS duration on the ECG before randomization” | **No target NP set**.  NT-proBNP of 1000 pg/mL was used as an inclusion criterion but patients were medically optimised at baseline | **Yes:** Increases of ≥30% in NT-proBNP compared with an individual NT-proBNP set-point | **More frequent** Visits at 1–3 month intervals to the HFC at the discretion of the investigators |
| **Shah** | **HF or cardiology clinic**: The trial was con- ducted at 3 centers with extensive experience in HF disease management: Duke | **Yes:** follow-up in the HF program of each site, and regular access to a telephone. | **Yes:** Before discharge, each patient received intensive HF education about medications and fluid and sodium management. | **Investigator led:**  BNP levels were 2 times greater than or less than the target BNP during clinic visits, investigators adjusted diuretic therapy according to a protocol and their clinical judgment. General guidelines for adjusting diuretics were offered to clinicians in a standard protocol. However, these were only guidelines, and the investigators were allowed to adjust diuretics as they deemed to be appropriate. | **Absolute** | **Less stringent.**  The discharge BNP served as the target BNP level for each individual patient. If BNP levels were 2 times greater than or less than the target BNP during clinic visits, investigators adjusted diuretic therapy according to a protocol and their clinical judgment. | **Relative**  If BNP levels were 2 times greater than or less than the target BNP during clinic visits, investigators adjusted diuretic therapy | **More Frequent:** Accordingly, patients were followed in the HF clinic at 1 week and 1, 2, 3, and 4 months after discharge. Clinicians |
| **Sochat** | **No specified:**  were followed for 16+-11 months in an outpatient clinic. | **None specified** | None specified | **Not clear:** | **Relative**: | **Less stringent:** Individual targets set | **Yes**: treatment was immediately intensified if NT- proBNP concentration was higher by more than 30% from previous measured level. | **More frequent:**  (every 45 +-19 days |
| **Skvortsov 2015** | **HF or cardiology clinic**: Hospital outpatients  (Dept. of myocardial diseases and heart failure, Russian Cardiology Research & Production complex) | **None specified** | None specified | **Pre-defined** “Drug therapy used in this study were based on practical recommendations for HF. There was a specific algorithm for intensification of therapy” | **Relative** decreases ≥50% from baseline. | **Stringent**  “The target value of NT-proBNP in patients in group therapy was <1000 pg/mL and / or decrease its ≥50% from baseline.” | **No** If NP increased without deterioration in clinical condition, patients received additional visit in 2 weeks. In case of increase of NT-proBNP concentrations with a parallel increase of symptoms corrective diuretic therapy was carried out immediately and individually for each patient. | **More frequent**: Every month up to 6 months, than every 3 months up to 1 year follow-up |
| **Troughton 2000** | **HF or cardiology clinic**: All patients received intensive follow-up every 3-months in a specialist heart-failure clinic (figure | **None specified** | None specified | **Pre-defined**: If these targets were not achieved, drug  treatment was intensified according to a strict and predetermined stepwise protocol | **Absolute** | **Less stringent:**  “Treatment target was clinically compensated heart failure N-BNP below 200 pmol/L (which corresponds to the concentration of BNP-32 that discriminated decompensated from compensated heart failure in an earlier study)” | **No:** If these targets were not achieved, drug treatment was intensified according | **More frequent**: Patients in either group not meeting treatment targets were reassessed at **2-week intervals** (by an investigator unaware of allocation) and treatment intensified (by the investigator who did know the allocation) until targets were met, at which point 3-month reviews were resumed. |

Table S2: Percentage of patients and the stage of New York Heart Association (NYHA) at study entry.

| Study | NYHA Stage 1 | NYHA Stage 2 | NYHA Stage 3 | NYHA Stage 4 |
| --- | --- | --- | --- | --- |
| Anguita | 0 | 0 | (n=42) 68% | (n=19) 32% |
| Berger | 0 | 0 | Unknown |  |
| Januzzi |  | 85% in this range | | 15%? |
| Jourdain |  | All in this range (median 2.2) | | 0% |
| Karlstrom | 0 | 30% | 55% | 14% |
| Krupicka |  | Mean 2.1 (+- 0.3) | | |
| Lainchbury | 8% | 67% | 23% | 2% |
| Schou | 85% in this range | |  |  |
| Skvortsov | 0 | 0 | (n=14) 24% | (n=44) 76% |
| Troughton |  | 72% |  |  |

## S2.

**Shrinkage Estimates for NP Meta-Analyses**

Graeme Spence

**Shrinkage Estimates**

- Reduce the variance of individual study estimates by using information from the whole meta-analysis.
- Uses Bayesian meta-analysis method to produce *shrinkage estimates*. Study estimates are ‘shrunk’ towards the pooled estimate.
- Can be useful when most favourable studies have the largest uncertainty.
- Requires random effects model – otherwise studies are assumed to have identical treatment effects (i.e. the fixed effects model).
- Hence, this method is not effective when little/no heterogeneity is observed.

## R code to fit shrinkage MA model.

BayesianMA <- function(event.t, n.t, event.c, n.c, model="BayesMA.bug",

t.mean=-4.25, t.var=1.56^2, n.iter=1E5){

# Calculate log RR effects and variance

meta <- metabin(event.e=event.t, n.e=n.t, event.c=event.c, n.c=n.c,

sm="RR", comb.fixed=F, comb.random=T)

Y <- meta$TE

v <- meta$seTE^2

# Calculate estimate for "typical" within study variance

ssq <- (length(Y) - 1) * sum(1/v) / ((sum(1 / v))^2 - sum(1/v^2))

# Set-up and run simulation

BayesMA.jags <- jags.model(model, n.chains = 3,

list(event.t=event.t, n.t=n.t,

event.c=event.c, n.c=n.c,

ssq=ssq, n=length(event.t),

t.mean=t.mean, t.prec=1 / t.var),

inits=list(theta=runif(1, -2, 2),

logit.isq=rt(1, 5) * sqrt(t.var * 3 / 5) + t.mean,

p.c=runif(length(Y),0,pmin(1,exp(-Y)))),quiet=TRUE)

update(BayesMA.jags, 100, progress.bar="none") #Burn-in

monitor=c("effect", "effect.i", "tsq")

coda.samples(BayesMA.jags, monitor, n.iter=n.iter,

thin=10, progress.bar="none")

}

BayesianMetaObject <- function(data, bayes.meta, label.left="", label.right=""){

# Standard meta-analysis

meta <- metabin(data=data, event.e=event.e, n.e=n.e, event.c=event.c, n.c=n.c, studlab=studlab, sm="RR", comb.fixed=F, comb.random=T)

# Obtain 2.5, 50 and 97.5% posterior quantiles from the Bayesian MA

posterior.quantiles <- summary(bayes.meta)$quantiles[1:(

dim(summary(bayes.meta)$quantiles)[1] - 1), c(1, 3, 5)]

# Duplicate meta-analysis and then override estimates and confidence intervals

BMA <- meta

BMA$lower <- log(posterior.quantiles[-1, 1])

BMA$TE <- log(posterior.quantiles[-1, 2])

BMA$upper <- log(posterior.quantiles[-1, 3])

BMA$lower.random <- log(posterior.quantiles[1, 1])

BMA$TE.random <- log(posterior.quantiles[1, 2])

BMA$upper.random <- log(posterior.quantiles[1, 3])

BMA

}

# Do the shrinkage analysis -----------------------------------------------

bma11 <- with(DT,BayesianMA(event.t = event.e, n.t = n.e, event.c = event.c, n.c = n.c, t.mean = -3.39, t.var = 1.84^2, n.iter = 1E6))

bnpdata <- DT[,c('Paper.name','event.e','n.e','event.c','n.c')]

names(bnpdata) <- c("studlab","event.e","n.e","event.c","n.c")

a <- BayesianMetaObject(bnpdata, bma11)

meta <- metabin(event.e=event.e, n.e=n.e, event.c=event.c, n.c=n.c,

sm="RR", comb.fixed=F, comb.random=T, data=bnpdata)

# output the forest plot

png(file="Figure 1.png", width = 2400,height = 1500,res = 350)

par(mfrow=c(1,1), mar=c(4,4.5,1,2.5), mgp=c(2,1,0))

forest(a, pooled.totals=F,

hetstat=F, lwd=1.5, lty.fixed=0, leftcols = "studlab",

rightcols = c("effect", "ci"), smlab="Shrinkage Estimates", just="center", label.left = "Favours NP", label.right = "Favours no NP",

text.random="Pooled effect", xlim=c(0.1,10))

dev.off()

**BayesMA.bug**

model {

for (i in 1:n) {

event.t[i] ~ dbin(p.t[i], n.t[i])

event.c[i] ~ dbin(p.c[i], n.c[i])

log(p.t[i]) <- log(p.c[i]) + min(log(effect.i[i]), -log(p.c[i]))

p.c[i] ~ dunif(0, 1)

effect.i[i] ~ dlnorm(theta, 1 / tsq)

}

theta ~ dnorm(0, 1 / 5)

logit.isq ~ dt(t.mean, t.prec, 5)

effect <- exp(theta)

tsq <- ssq * exp(logit.isq)

}

Figure S2: Forest plot showing “shrinkage estimates” of the relative risk of all-cause mortality.


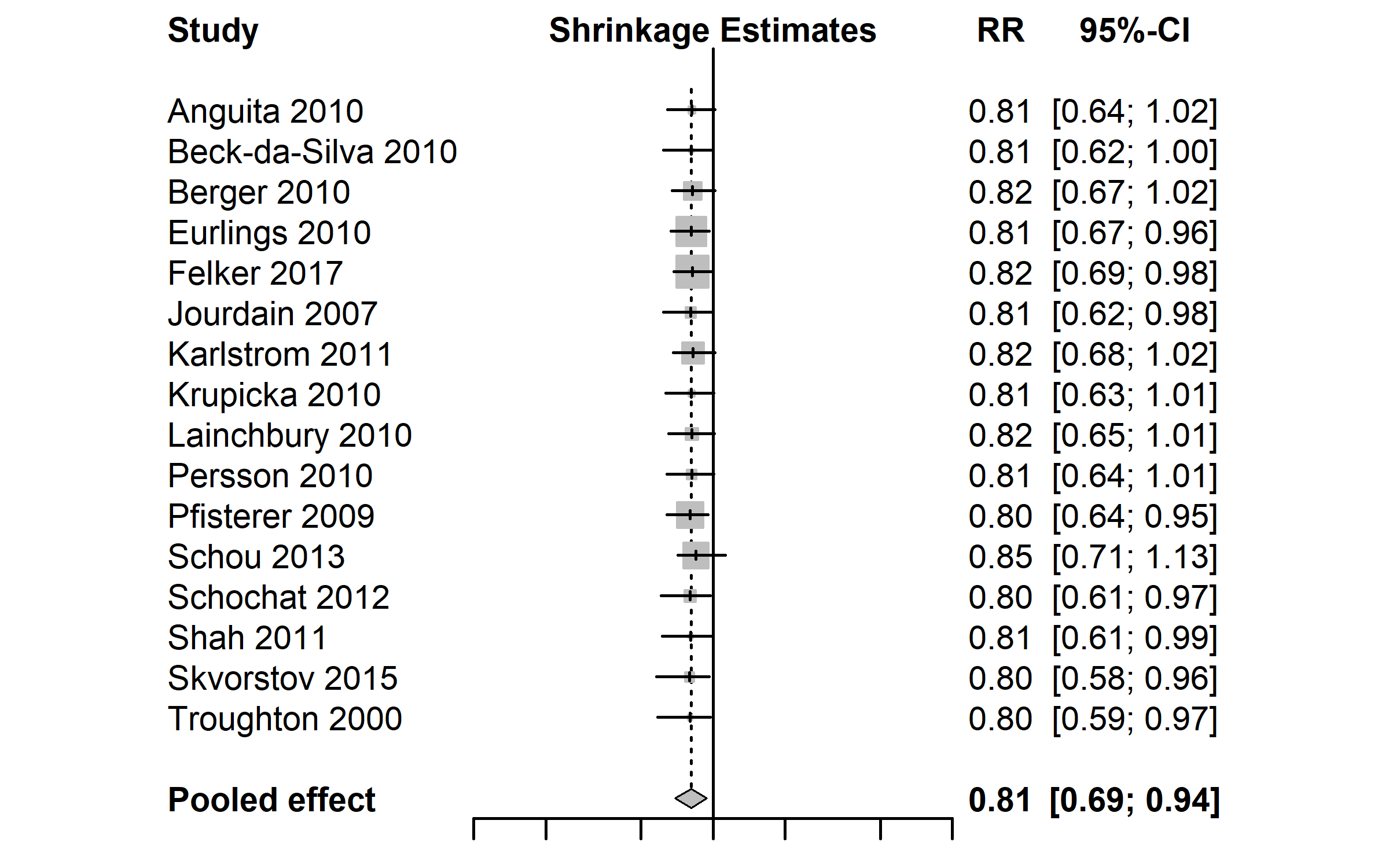


## Figure S3: Study flow-chart.

##

Li JJ, Xiang XL, Tian XY, Shi YF. Clinical research on brain natriuretic peptide guiding the application of beta1 receptor blocker in patients with moderate to severe heart failure. Acta Cardiologica Sinica 2015; 31(1):52-8.

Maeder MT, Rickenbacher P, Rickli H, Abbu H, Gutmann M, Erne P, et al. N-terminal pro brain natriuretic peptide-guided management in patients with heart failure and preserved ejection fraction: findings from the Trial of Intensified versus standard Medical therapy in Elderly patients with Congestive Heart Failure (TIME-CHF). European Journal of Heart Failure 2013; 15:1148-56
